# Partial Sperm beta1 Integrin Subunit Deletion Proves Its Involvement in Mouse Gamete Adhesion/Fusion

**DOI:** 10.3390/ijms21228494

**Published:** 2020-11-11

**Authors:** Virginie Barraud-Lange, Côme Ialy-Radio, Céline Chalas, Isabelle Holtzmann, Jean-Philippe Wolf, Sandrine Barbaux, Ahmed Ziyyat

**Affiliations:** 1 Institut Cochin, Université de Paris, INSERM, CNRS, F-75014 Paris, France; virginie.barraud-lange@aphp.fr (V.B.-L.); come.ialy-radio@inserm.fr (C.I.-R.); celine.chalas@aphp.fr (C.C.); isabelleholtzmann@hotmail.com (I.H.); jean-philippe.wolf@aphp.fr (J.-P.W.); sandrine.barbaux@inserm.fr (S.B.); 2 Service d’Histologie, d’Embryologie, Biologie de la Reproduction, AP-HP, Hôpital Cochin, F-75014 Paris, France

**Keywords:** fertilization, sperm, integrin

## Abstract

We have previously shown, using antibodies, that the sperm alpha6beta1 integrin is involved in mouse gamete fusion in vitro. Here we report the conditional knockdown of the sperm *Itgb1* gene. It induced a drastic failure of sperm fusogenic ability with sperm accumulation in the perivitelline space of in vitro inseminated oocytes deleted or not for the *Itgb1* gene. These data demonstrate that sperm, but not oocyte, beta1 integrin subunit is involved in gamete adhesion/fusion. Curiously, knockdown males were fertile in vivo probably because of the incomplete Cre-mediated deletion of the sperm *Itgb1* floxed gene. Indeed, this was shown by Western blot analysis and confirmed by both the viability and litter size of pups obtained by mating partially sperm *Itgb1* deleted males with females producing completely deleted *Itgb1* oocytes. Because of the total peri-implantation lethality of *Itgb1* deletion in mice, we assume that sperm that escaped the *Itgb1* excision seemed to be preferentially used to fertilize in vivo. Here, we showed for the first time that the deletion, even partial, of the sperm *Itgb1* gene makes the sperm unable to normally fertilize oocytes. However, to elucidate the question of the essentiality of its role during fertilization, further investigations using a mouse expressing a recombinase more effective in male germ cells are necessary.

## 1. Introduction

Oocyte integrins have been considered as mandatory to enable a correct gamete interaction [1] before their role was questioned by deletion experiments showing that normal fertilization occurs with eggs lacking either α6 or β1 integrin subunits both in vivo and in vitro [2,3]. However, in these experiments, the sperm used was obtained from wild type (WT) mice. In parallel, we have demonstrated that α6β1 integrin is actually present on the sperm membrane and is involved in the sperm-egg fusion process [4]. Therefore, experimental results and literature data led us to hypothesize that the sperm β1 integrin could compensate for the loss of oocyte β1 integrin.

Indeed, using preincubation protocols of sperm, oocytes or both gametes, with anti-integrin antibodies before insemination of cumulus-enclosed oocytes, we have shown that inhibition of sperm integrins significantly reduces fertilization rate (FR). Furthermore, in the zona-free oocyte model, sperm-egg fusion is inhibited by sperm preincubation with anti-integrin antibodies suggesting that adhesion/ fusion is actually a step where sperm integrins are involved [4,5].

Furthermore, the expression of adhesion molecules on spermatozoa from several species has already been reported. The β1 integrin subunit has been detected by histochemistry on the basement membrane of tubuli seminiferi, spermatocytes, spermatids and testicular spermatozoa in human [6]. A positive correlation between the expression of β1 integrin on human spermatozoa and their fertilizing ability has been demonstrated, which suggests that sperm integrins may be a putative determinant in egg-sperm recognition and interaction [7,8]. Hence, Reddy et al. proposed α6β1 as a clinical marker to evaluate sperm quality [7]. Furthermore, β1 integrin forms heterodimers with other α integrin subunits than α6. This is the case of α3β1 expressed at the level of the sperm outer acrosomal membrane [9]. Similarly, other β integrin subunits are also expressed on the sperm head. Recently, using super-resolution microscopy accompanied by colocalization analysis, Frolikova et al. located integrin α6β4 in the inner apical acrosomal membrane and equatorial segment [9]. Sperm expression of β3 integrin subunit has been reported to be similar to that of the αv integrin subunit; the percentage of sperm cells displaying β3 integrin subunit being correlated with the proportion of spermatozoa that had undergone an acrosome reaction (AR) following ionophore exposure. This suggests their implication in this function [10]. α5β1 integrin (fibronectin receptor) and αvβ3 integrin (vitronectin receptor) became apparent on sperm surface during capacitation and following the AR, respectively [10]. Other studies performed on humans demonstrated correlations between integrin expression level and fertilization ability [11,12,13]. Another study performed in bovine with separately preincubated gametes, has also shown that either the RGD peptide or the anti-αv and/or anti-α5 antibodies inhibit fusion whatever was the incubated gamete [14,15]. The inhibition induced by exogenous supplementation with fibronectin seemed to be exerted on the male gamete by binding to the exposed integrin α5β1 receptor after AR [16]. More recently, it has been found that fibronectin, via sperm α5β1 integrin, induced capacitation-associated events [17]. Other investigations have shown that α6β1 integrin is present on boar sperm membrane and that sperm accumulate this molecule on the membrane and concentrate it on the acrosomal region as capacitation progresses [18,19]. More generally, the relocation of sperm surface proteins, particularly after AR, seems to be critical. We very recently observed the relocation of SPACA6 during AR from the acrosomal cap region to the equatorial segment, where fusion initially takes place [20]. Such a relocation had already been observed for IZUMO1 [21,22,23]. Interestingly, it has been shown that in the absence of the TSSK6 kinase, the relocation of IZUMO1 during the AR, from the acrosomal cap region to the equatorial segment, is not done and consequently sperm are unable to fertilize [24]. No less interestingly, it has been shown that β1 integrin relocates across the apical equatorial segment towards the whole equatorial segment and the whole sperm head during AR [25].

Since adhesion proteins seem widely involved in sperm-egg interaction and to further describe the function of sperm integrins and understand their role in gamete interaction, we performed a conditional knockout (KO) of sperm *Itgb1* that turned out to be a conditional knockdown (KD) rather than KO. In vitro, sperm of KD mice were severely handicapped in their ability to fertilize and many were stored within the perivitelline space indicating their inability to fuse normally with the oolemma. However, these males were fertile in vivo, suggesting the existence of compensatory mechanisms in the natural fertilization process.

## 2. Results

### 2.1. Cre-Mediated Deletion of the Itgb1 Gene in Oocytes and Sperm

**Figure 1:**
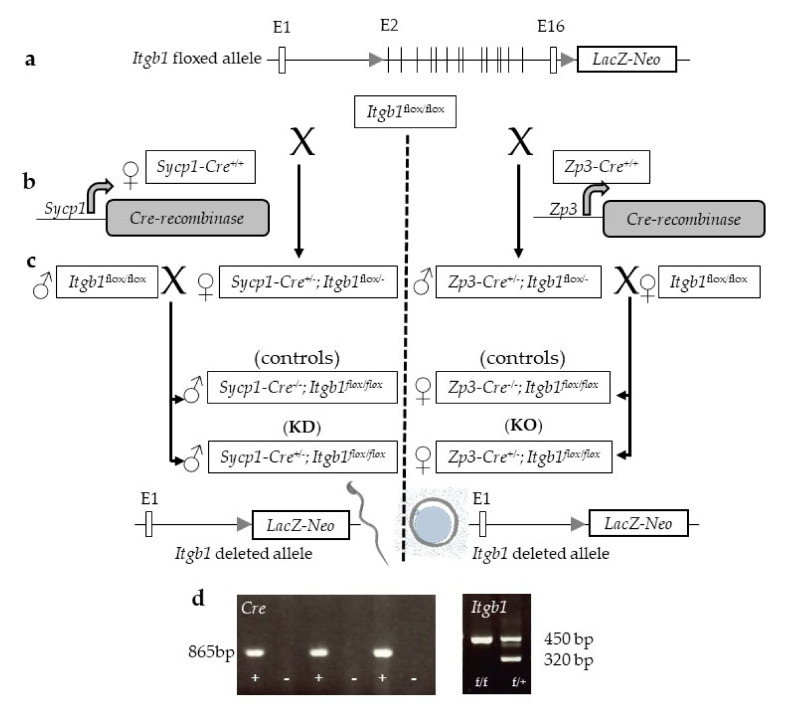
(**a**) Schematic representation of the *Itgb1* floxed allele. To monitor Cre mediated deletion, a promoterless *LacZ* gene was inserted downstream of the 3′ loxP site, resulting in *LacZ* expression driven by the endogenous *Itgb1* promoter only after deletion; (**b**) First round mating using *Itgb1 ^flox/flox^* with *Sycp1-Cre ^+/+^* females or *Zp3-Cre ^+/+^* males to obtain double heterozygous *Sycp1-Cre ^+/−^ Itgb1 ^flox/-^* or *Zp3-Cre ^+/−^ Itgb1 ^flox/−^* that (**c**) we mated, respectively, with *Itgb1 ^flox/flox^* males or females. These crosses made it possible to obtain (left) the males of interest (*Sycp1-Cre ^+/−^ Itgb1 ^flox/flo^*, KD) and their controls (*Cre ^−/−^ Itgb1 ^flox/flox^*) and (right) the females of interest (*Zp3-Cre ^+/−^ Itgb1 ^flox/flox^*, KO) and their controls (*Zp3-Cre ^−/−^ Itgb1 ^flox/flox^*); (**d**) By way of example, the two gels show the presence of *Cre* whether it is under the control of the *Zp3* promoter or that of *Sycp1* promoter and the presence of the Lox sites on one or two alleles.

Figure 1 illustrates the crossing scheme used in order to obtain respectively the males or the females in which the sperm or the oocytes are invalidated for the *Itgb1* gene. Homozygous floxed *Itgb1* gene mice without Cre expression were used as controls and homozygous floxed *Itgb1* gene mice expressing Cre represented the mice of interest to be tested.

**Figure 2:**
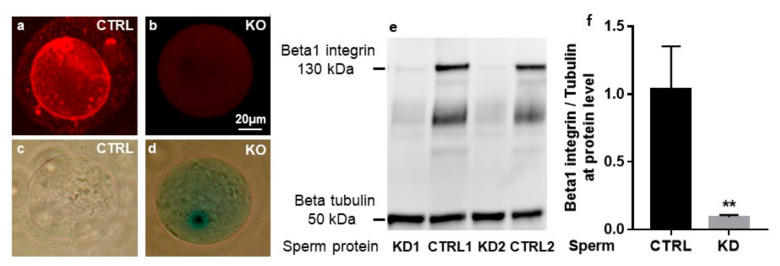
Analysis of the excision of the *Itgb1* floxed gene in the oocytes and the sperm. Analysis of immunofluorescence staining of β1 integrin in control oocytes (CTRL) (**a**) and knockout (KO) (**b**) oocytes using the anti-β1 integrin monoclonal antibody (MB1.2). Analysis of β-galactosidase staining in CTRL (**c**) and KO (**d**) oocytes. (**e**) immunoblotting with anti-β1 integrin and anti-β-tubulin antibodies was performed as described in Materials and Methods. The specific bands of β1 integrin subunit and β-tubulin (130 kDa and 50 kDa, respectively) were detected on sperm extracts. Analysis showed decreased expression levels in KD sperm (lanes KD1 and KD2) when compared to CTRL sperm (CTRL1 and CTRL2) while the level of expression of β-tubulin was comparable in the four samples. (**f**) quantification of Western blot band intensities using ImageJ software. The detection of β-tubulin in each sample served as a loading control. The relative intensities of the protein signals were quantified by densitometry and normalized to the corresponding β-tubulin density. Data are expressed as percentage relative to CTRL. The bar graphs represent the mean ± s.e.m. of 3 samples in each group. ** *p* = 0.005.

As evaluated by immunofluorescence using a rat anti-mouse β1 integrin monoclonal antibody (MB1.2), all the ovulated oocytes from conditional KO mice (*Zp3-Cre ^+/−^ Itgb1 ^flox/flox^*, Figure 2b) contrary to control oocytes (CTRL, *Zp3-Cre ^−/−^ Itgb1 ^flox/flox^*, Figure 2a) showed no staining as previously documented (Figure 2a,b) [3]. Supporting this result, deleted oocytes expressed the *LacZ* gene under the control of the *Itgb1* promoter (Figure 2d) contrary to CTRL oocytes (Figure 2c).

Western blot analyses of sperm from *Sycp1-Cre ^+/−^ Itgb1 ^flox/flox^* mice (KD1 and KD2) demonstrated weak but not absent signals corresponding to β1 integrin compared to the control sperm from *Sycp1-Cre ^−/−^ Itgb1 ^flox/flox^* (CTRL1 and CTRL2, Figure 2e) while β-tubulin expression was comparable in all samples. This result indicated that the Cre recombinase was not totally effective. We used ImageJ software to measure the intensity of the bands corresponding to β1 integrin normalized by β-tubulin expression levels. The comparison revealed that the expression level of β1 integrin in KD sperm represented 14.1 ± 10.8% of the WT level (Figure 2f, *p* = 0.0055).

### 2.2. In Vivo and in Vitro Evaluation of the Fertilizing Ability of Sycp1-Cre ^+/−^ Itgb1 ^flox/flox^ Sperm and Zp3-Cre ^+/−^ Itgb1 ^flox/flox^ Oocytes

**Figure 3:**
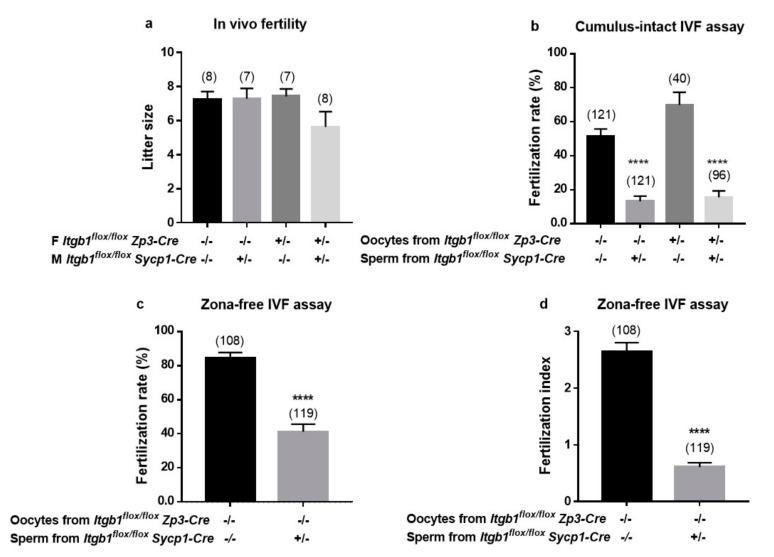
In vivo and in vitro analysis of the fertilizing ability of *Sycp1-Cre ^+/−^ Itgb1 ^flox/flox^* males (M) and *Zp3-Cre ^+/−^ Itgb1 ^flox/flox^* females (F). (**a**) histogram representing the mean litter size of conditional KO (*Zp3-Cre ^+/−^ Itgb1 ^flox/flox^*) or control (*Zp3-Cre ^−/−^ Itgb1 ^flox/flox^*) females when mated with conditional knockdown (KD) (*Sycp1-Cre ^+/−^ Itgb1 ^flox/flox^*) or control (*Sycp1-Cre ^−/−^ Itgb1 ^flox/flox^*) males, respectively (the numbers in parentheses represent the number of litters in each group). No statistical difference was revealed between the different groups (*p* = 0.3); (**b**) fertilization rate (FR) (mean ± s.e.m.) following cumulus-intact in vitro fertilization (IVF) assay at 10^6^ spermatozoa per ml for 3 h. Studies were repeated four times. All males and females were homozygously floxed for *Itgb1* gene. The heterozygous presence and expression of the *Cre* gene in the sperm or oocytes determined the deletion status (KO or KD). The mean FR for *Itgb1* KO eggs were 16.5% ± 3.3% and 69.2% ± 7.5% (*p* < 0.0001) depending on whether the sperm used was from Itgb1 KD males or control, respectively. The mean FR for control eggs were 12% ± 4.6% and 51.2% ± 4.6% (*p* < 0.0001) depending on whether the sperm used was from *Itgb1* KD males or control, respectively; (**c**,**d**) FR and fertilization index (FI) (mean ± s.e.m.) following zona-free IVF assay at 10^5^ spermatozoa per ml for 3 h were presented, respectively. The FR dropped from 84.2 ± 3.5 to 41.1 ± 4.5 ((**c**) *p* < 0.0001) and the FI dropped from 2.6 ± 0,1 to 0.6 ± 0.07 ((**d**) *p* < 0.0001) when wild type (WT) oocytes were inseminated with sperm from control or KD males, respectively. Studies were repeated four times; In (**b**–**d**) the numbers in parentheses represent the number of used oocytes analyzed in each group.

The different matings performed between mutated and/or control mice showed no difference in fertility between couples (Figure 3a). Since *Itgb1* total deletion in mice results in inner cell mass failure and total peri-implantation lethality, as it has been shown by two different KO mice [26,27], the development of pups demonstrates, for sure, that *Itgb1* deleted oocytes were necessarily fertilized by a sperm containing a non-deleted *Itgb1* allele. This indicates that the non-deleted sperm fertilize predominantly.

As for in vivo, in in vitro experiments, *Sycp1-Cre ^−/−^ Itgb1 ^flox/flox^* and *Zp3-Cre ^−/−^ Itgb1 ^flox/flox^* mice were taken as controls. When *Itgb1* KD sperm were used, the FR in cumulus-intact oocytes dropped from 51.2 ± 4.5% to 13.2 ± 3.0% (*p* < 0.0001). By contrast, deletion of oocytes *Itgb1* did not significantly change the FR (70.2 ± 7.3% for deleted oocytes vs. 51.2 ± 4.5% for control oocytes). Finally, when *Itgb1* was deleted for both male and female gametes, the FR was 15.6 ± 3.7% (not significantly different from the situation where only sperm was deleted, 13.2 ± 3.0%; *p* = 0.6) (Figure 3b). These results indicate that the oocyte β1 integrin subunit is not essential for the gamete adhesion/fusion process and that only the sperm β1 integrin subunit is important for a successful gamete interaction, at least in vitro. This difference in term of FR was confirmed in zona-free assays since we obtained a FR of 84.2 ± 3.5% and 41.1 ± 4.5% when control oocytes were inseminated with control or KD sperm, respectively (*p* < 0.0001) (Figure 3c). This difference was also found in terms of fertilization index (FI: number of fused sperm per zona-free oocyte), going from 2.63 ± 0.16 for the control group to 0.60 ± 0.07 for the group using *Itgb1* KD sperm (*p* < 0.0001) (Figure 3d).

### 2.3. In vitro Accumulation of Sperm from Sycp1-Cre ^+/−^ Itgb1 ^flox/flox^ Males in the Perivitelline Space of the Oocytes

**Figure 4:**
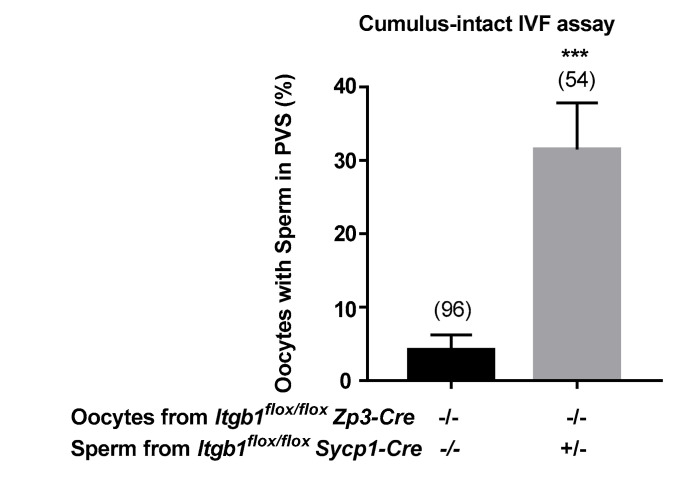
Sperm accumulation in oocytes’ perivitelline space (PVS) after control oocytes (*Zp3-Cre ^−/−^ Itgb1 ^flox/flox^)* insemination with sperm from KD (*Sycp1-Cre* ^+*/−*^ *Itgb1 ^flox/flox^*) or control (*Sycp1-Cre* ^−*/−*^ *Itgb1 ^flox/flox^*) males. After cumulus-intact IVF assays, was reported the percentage of PVS containing-oocytes (mean ± s.e.m.) that showed high and significant difference when comparing the two groups (*** *p* < 0.0001). The numbers in parentheses represent the number of used oocytes in each group.

Because the adhesion and fusion of the spermatozoon, that penetrated into the perivitelline space (PVS), with the oolemma prevents subsequent sperm penetration, sperm can rarely be found in the PVS when WT sperm are used in fusion assay. By contrast, when *Itgb1* KD sperm from *Sycp1-Cre* ^+*/−*^ *Itgb1 ^flox/flox^* males were used, an accumulation of many sperm (from 1 to up to 15 per oocyte) inside the PVS was observed (Figure 4) in about one third of the oocytes (31.4% ± 6.3%), indicating that they were not competent for membrane fusion and/or that they necessitate a delay to fuse. Statistical analysis on the number of oocytes with sperm into the PVS showed a very significant difference between oocytes inseminated with even partially deleted *Itgb1* sperm (31.5 ± 6.4%) and those inseminated with *Sycp1-Cre* ^−*/−*^ *Itgb1 ^flox/flox^* control sperm (4.1 ± 2.0%; *p* < 0.0001; Figure 4).

## 3. Discussion

Oocyte α6β1 integrin has been described as the sperm receptor [1], but this role was challenged by deletion experiments [2,3]. Then, we have shown that this α6β1 integrin is in fact also expressed by sperm and actually involved in gametes interaction [4]. To study its functions in vivo, we tried to generate *Itgb1* sperm conditional KO mice. Due to the partial efficiency of the Cre activity under the control of the promoter of the male germ cells specific gene, *Sycp1*, the mouse line obtained was actually only a KD. Indeed, others have also reported the incomplete efficiency of the *Cre* recombinase under the control of the *Sycp1* promoter [28]. When the mice were produced during multiple generations, the LoxP sites progressively fail to allow recombination due to accumulating epigenetic modifications. It has also been recommended to transfer the Cre activity exclusively through the female germ line to avoid a transvection phenomenon susceptible to prevent Cre access to its specific LoxP site. This phenomenon occurs when Cre protein and LoxP sites coexist in the same cell during male meiosis [28,29]. Despite the respect of this protocol, we still found a residual amount of β1 integrin in the sperm. By estimating the amount of β1 integrin protein on KD sperm by Western analysis (about 15% compared to that from control sperm), we indirectly assessed the proportion of non-deleted sperm. As sperm develop in a syncytium wherein germ cells are connected by cytoplasmic bridges through which mRNA and protein are shared, we think that the percentage of sperm containing the integrin β1 protein is greater than that obtained by estimating the quantity of proteins. The concept of genetically different but phenotypically equivalent spermatids had been demonstrated several decades ago using hemizygous transgenic mice [30]. Anyhow, the persistence of a proportion of sperm, even small, that expressed β1 integrin, seemed to ensure an almost normal fertility in vivo. The use of females in which oocytes were deleted for *Itgb1* allowed us to conclude that first: The oocyte β1 integrin subunit is not mandatory for fertilization, confirming previously published data [3] and second: That the non-deleted *Itgb1* sperm is preferably used to fertilize in vivo. It should be noted however that this group of mating between females with KO oocytes and males with KD sperm gave slightly smaller, but not significant, litter sizes than the other groups. This small difference could be explained by the peri-implantation mortality of embryos totally invalidated for *Itgb1*. Indeed, *Itgb1* invalidation in mice results in inner cell mass failure and total peri-implantation lethality as it has been shown by two different KO mice [26,27]. We can affirm this conclusion because immunofluorescence analysis showed a total lack of β1 integrin on the surface of deleted oocytes as also reported before [3]. In vitro, our data are also in agreement with those reported by Miller et al. and He et al. stating that oocyte integrins are not essential for gamete adhesion/fusion [2,3]. The fact that we observed no effect related to the absence of oocyte β1 integrin subunit does not mean however that it does not participate in the fertilization process. Using anti-integrin antibodies, we have already shown a significant inhibition of the FR after oocytes preincubation [4]. Such inhibition was found using another anti-β1 integrin antibody in conditions of low sperm: egg ratios [31]. However, in this last study, as in others, the antibody was present during in vitro fertilization (IVF) and its action on sperm integrin cannot be excluded. An experiment, using RNAi, could have demonstrated the role of oocyte β1 integrin, but no conclusion could be drawn because the level of protein expression remained unchanged at the oolemma [31]. The lack of an inhibitory effect in the absence of oocyte integrin in our study could be explained by several reasons. A high sperm concentration (1x10^6^ sperm/mL) was used in our experimental conditions compared to that used in the above-mentioned study [31]. The level of action (gene, RNA or protein) certainly does not cause the same adaptive responses. Thus, the deletion of the gene may be accompanied by the overexpression of another gene playing a redundant role. Such a response cannot occur when using an antibody or RNAi. Indeed, a very demonstrative example published by Evans et al. [32] showed that α9 integrin, that has been reported to dimerize only with the β1 integrin subunit, is able, in the absence of the latter, to dimerize with the β7 integrin subunit. This compensatory dimerization encourages a cautious interpretation of the lack of strong phenotype after gene deletion experiments. Another explanation comes from a study in which the authors have determined that polymers displaying a peptide from the fertilin β disintegrin domain mediate inhibition of mammalian fertilization only when a β1 integrin receptor was present on the egg surface (WT oocytes). Such inhibition did not occur when the β1 integrin was absent from the egg surface (KO oocytes). They concluded that the mechanisms by which sperm fertilize WT and β1 integrin KO eggs are different [33]. The role of oocyte β1 integrin remains debatable, but typically the fact that a molecule is not essential to a function does not exclude that it may participate in this function. 

In our study, we focused on sperm integrin. The absence of sperm β1 integrin, even if it was not total, has led to a drastic decrease in the fertilization rate and index whether the oocytes used were control or KO for *Itgb1*, highlighting the much more important role played by sperm β1 integrin.

The other important phenotype that we have observed is that of the presence of sperm in the PVS. Indeed, when fertilization occurs normally, the first sperm that fuses triggers the cortical reaction, itself responsible of the polyspermy block at the Zona pellucida (ZP) level. The presence of supernumerary sperm in the PVS means that the first sperm that entered into the PVS (and sometimes even the following ones) is unable or at least has to struggle to fuse with the oocyte membrane. This phenotype is extremely important since it is common to all mouse models of genes invalidation considered as essential for gamete adhesion/ fusion. A similar phenotype was first observed in *Cd9* null oocytes [34,35], and more recently in *Juno* null oocytes ([36]. Furthermore, in WT oocytes inseminated in vivo or in vitro with sperm that lack *Izumo1*, *Spaca6, Sof1, Tmem95* or *Fimp* [20,37,38,39]. Interestingly, the fertilization failure was not due to a failure to penetrate the ZP because sperm have entered and accumulated into the PVS. We therefore concluded that β1 integrin is not involved in binding to and penetrating through the cumulus cells and ZP. This finding emphasizes the important role of sperm β1 integrin in gamete membrane interaction. Then to prove the essential character of this role, a mouse with a more efficient Cre than that expressed under the control of *Sycp1* is necessary. Mice like *Stra8-Cre* [40] or *Ngn3-Cre* [41] could help answer this question.

The discrepancy between the in vivo and in vitro results with respect to the ability of deleted sperm to fertilize oocytes is not fully explained. Nevertheless, hypotheses can be proposed. This difference could be probably, at least partially, explained by the optimal conditions and the high efficiency of in vivo fertilization compared to in vitro fertilization. Therefore, IVF can better reveal even minor functional alterations. If evidence was needed, the difference of the number of sperm needed to fertilize oocytes in vivo or in vitro is very large. Indeed, while it is estimated that the 100 to 200 sperm that succeed to reach the ampullae in vivo are sufficient to fertilize all the oocytes, even after superovulation (between 20 and 30 per mouse), one million sperm per ml (10^5^ in 100 µL of medium drop) are necessary for a poorer rate of in vitro fertilization of a similar oocyte pool.

Otherwise, the fact that the litter sizes were comparable after mating of WT females with WT or sperm *Itgb1* conditional KD males suggests that even in the later situation, the number of sperm bearing β1 integrin subunit around the cumulus oophorus complex could be normal. In other words, it appears that sperm bearing β1 integrin subunit could be selected during their ascent through the female genital tract. Such hypothesis would imply that β1 integrin subunit is important for the sperm to migrate through the female genital tract. If the sperm migrated into the female genital tract irrespective of their status (expressing β1 integrin or not), we would have had, in vivo, a situation similar to that observed in vitro with sperm competition in favor of the most numerous sperm type, i.e., deleted ones. This should have resulted in a lower fertilization rate and smaller litter sizes, contrary to what we observed. One can therefore hypothesize that β1 integrin subunit could be involved in sperm-epithelium binding within the “sperm reservoir”. Indeed, during their transit through the female genital tract, spermatozoa transiently bind to epithelial cells of the caudal isthmus leading to the concept of “sperm reservoir”. This binding has been described in several species, including mice [42] and humans [43]. However, the identity of the molecular players that mediate sperm-oviduct adhesion is still largely mysterious. Nevertheless, infertility phenotypes due to a defect of sperm migration in the female genital tract have already been reported in the absence of an adhesion molecule such as ADAM3 (A Disintegrin and A Metalloprotease 3) [44]. Furthermore, like ADAMs, integrins are cell–extracellular matrix and cell–cell adhesion molecules. According to our hypothesis, sperm containing β1 integrin subunit could preferentially reach the fertilization site making the situation where fertilization takes place similar to the control one.

Despite the difficulty to show a phenotype in vivo, (i) the drastic decline of fertilization rate and index in vitro after cumulus-intact and zona-free assays, (ii) the presence of sperm into the PVS and (iii) finally the fact that mice from the mating between oocyte knockout females and sperm knockdown males survived is a body of evidence that seems sufficient to affirm the important role of sperm beta1 integrin in sperm-egg adhesion/fusion. In conclusion, we showed for the first time that deletion, even partial, of the sperm *Itgb1* makes the sperm unable to normally fertilize oocytes. 

## 4. Materials and Methods 

### 4.1. Ethics Statement

All animal experiments were performed in accordance with national guidelines for the care and use of laboratory animals. Authorizations were obtained from local (C2EA-34, Comité d’éthique en matière d’expérimentation animale Paris Descartes; 1 February 2013) and governmental ethical review committees via APAFiS Application (Autorisation de projet utilisant des animaux à des fins scientifiques), Authorization APAFIS #14124-2017072510448522 v26, A. Ziyyat (2018–2023).

### 4.2. Generation of Oocyte and Sperm Itgb1 Conditional Knockout Mice

These mice were generated as previously described [3]. The floxed *Itgb1* gene mice have been provided by S. Dufour (Institut Curie, Paris, France) according to the agreement of R. Fassler (Max Planck Institute, Martinsried, Germany). To monitor Cre-mediated deletion, a promoterless *LacZ* gene was inserted downstream of the 3′ LoxP site, resulting in *lacZ* expression driven by the endogenous *Itgb1* promoter. Transgenic mice expressing the *Cre* recombinase under the control of the *Zp3* promoter from the Jackson laboratory were mated with the floxed *Itgb1* mice. Heterozygous mice for floxed *Itgb1* and *Zp3-Cre* were mated with floxed *Itgb1* mice. Female mice with both the *Zp3-Cre* transgene and homozygous floxed *Itgb1* gene (*Zp3-Cre ^+/−^ Itgb1 ^flox/flox^*) were used for in vivo mating. Eggs collected from these mice were used for β1 integrin subunit immunofluorescent staining, beta-galactosidase detection and IVF assays.

To generate sperm *Itgb1* conditional KD male mice, the same procedure was performed but using *Sycp1-Cre* mice. Male mice with the *Sycp1-Cre* transgene and homozygous floxed *Itgb1* gene (*Sycp1-Cre ^+/−^ Itgb1 ^flox/flox^*) were used for in vivo mating and IVF assays. For all experiments, controls used were *Sycp1*- or *Zp3*- *Cre ^−/−^ Itgb1 ^flox/flox^* males or females, respectively.

Genotyping of mice was performed by PCR amplification (GoTaq^®^ DNA Polymerase, Promega, Madison, WI, USA) on DNA extracted from tail biopsies (NucleoSpin^®^ Tissue, Macherey-Nagel, Düren, Germany) using the following primers respectively: 5′-AGGTGCCCTTCCCTCTAGA-3′ and 5′-GTGAAGTAGGTGAAAGGTAAC-3′ for floxed *Itgb1* gene detection [45] and one or the other of these two pairs of primers: 5′-TGATGGACATGTTCAGGGATC-3′ and 5′-CAGCCACCAGCTTGCATGA-3′ or 5′-GCGGTCTGGCAGTAAAAACTATC-3′ and 5‘-GTGAAACAGCATTGCTGTCACTT-3′ for *Cre* gene detection under the control of *Zp3* or *Sycp1* promoters. The first primers pair gives an amplification of 320 bp for WT *Itgb1* and 450 bp for floxed *Itgb1.* The second and the third ones give amplimers of 865 bp [46] or 100 bp [47], respectively. 

### 4.3. Immunofluorescence and Beta-galactosidase Staining of Mouse Oocytes

Zona-intact cumulus-free oocytes from control or conditional KO mice were fixed during 20 min in 4% formaldehyde at room temperature (RT) and washed three times in PBS-BSA 1%. For detection of the β1 integrin subunit, oocytes were incubated with the primary antibody (MB1.2 at 20 µg/mL; Chemicon International, Temecula, CA, USA) for 1 h at RT followed by Alexa Fluor^®^ 594 goat anti-rat IgG secondary antibody incubation (10 µg/mL, Molecular Probes, Invitrogen, Illkirch, France). Control immunofluorescent studies were performed using isotype (IgG2a) (Serotec-Bio-Rad, Marnes-la-Coquette, France) as primary antibody or Alexa Fluor^®^ 594 goat anti-rat IgG alone. Oocytes were washed and directly mounted in Vectashield (Vector Laboratories, Burlingame, CA, USA) and covered with a coverslip for analysis. Detection was performed using a Zeiss Axiophot epifluorescence microscope and images were digitally acquired with a camera (Coolpix 4500, Nikon).

For β-galactosidase staining, oocytes were transferred into freshly prepared X-Gal staining solution according to the manufacturer’s instructions (Roche, Mannheim, Germany), and stained for 30 min at 37°C. After rinsing with PBS-BSA 1%, oocytes were examined under light microscopy.

### 4.4. Western Blot Analysis and Quantification of Sperm β1 Integrin

Capacitated WT or β1 KD sperm were washed twice in PBS, the pellet was snap-frozen in liquid N_2_ and stored at –80 °C for further use. Sperm aliquots (2 × 10^6^) were lysed in 50 mM Tris (pH 8), 150 mM NaCl, 1 mM EDTA, 0.25% sodium deoxycholate and 1% NP40, supplemented with Protease Inhibitor Cocktail (Sigma, St. Louis, MO, USA) for 1 h on ice, gently sonicated with an equal volume of 2x NuPAGE^®^ LDS sample buffer (Thermo Fisher Scientific, Illkirch, France). Protein concentrations were determined by microBCA (Pierce, Thermo Fischer Scientific, Illkirch, France) and sperm proteins were separated by electrophoresis in Novex^®^ Tris-glycine precast gel (Thermo Fisher Scientific, Illkirch, France) and electro-transferred to Immobilon-P membranes (GE Healthcare, France). Membranes were blocked for 1 h with 2% casein prior to incubation with primary anti-β1 integrin antibody at 1:250 for 90 min at 37°C, and anti-β-tubulin antibody (Merck-Millipore, Molsheim, France) at 1:15 000 for 90 min at RT followed by appropriate secondary HRP conjugated antibodies (0.2 µg/mL) at RT. HRP activity was revealed by ECL detection kit (Merck-Millipore, Molsheim, France) and ImageQuant LAS 4000 for quantitative imaging.

### 4.5. In Vivo Mating, Gamete Preparation and in Vitro Fertilization

Four groups of mating were used: KD males (i.e., males expressing *Cre* under the control of the *Sycp1* promoter at the heterozygous state) with KO females (i.e., females expressing *Cre* under the control of the *Zp3* promoter at the heterozygous state), KD males with control females, control males with KO females and control males with control females. All mice were homozygous for the *Itgb1* floxed gene. Mice were aged from 8 to 12 weeks and were housed as one male and one female per cage. In each group, litter size was assessed and compared to other groups.

### 4.6. Gamete Preparation and in Vitro Fertilization

WT and conditional KO female mice (5–8 week-old) were superovulated with 5 IU PMSG (pregnant mare serum gonadotropin) and 5 IU hCG (human chorionic gonadotropin) (Intervet, Beaucouze, France) 48 h apart. Fourteen hours after hCG injection, animals were sacrificed by cervical dislocation. Cumuli oophori were collected by tearing the ampulla wall of the oviduct, placed in Ferticult medium (FertiPro, Beernem, Belgium) supplemented with 3% BSA, and maintained at 37 °C under 5% CO_2_ in air under mineral oil (Sigma, St. Louis, MO, USA) For zona-free IVF assay, oocytes were freed from the cumulus cells by 3–5 min incubation at 37 °C with hyaluronidase (Sigma, St. Louis, MO, USA) in M2 medium (Sigma, St. Louis, MO, USA) Oocytes were rinsed and kept in Ferticult medium at 37 °C under 5% CO_2_ atmosphere under mineral oil (Sigma, St. Louis, MO, USA). ZP was then dissolved with acidic Tyrode’s (AT) solution (pH 2.5, Sigma, St. Louis, MO, USA) under visual monitoring. The zona-free eggs were rapidly washed in medium and kept at 37 °C under 5% CO_2_ atmosphere for 2 to 3 h to recover their fertilizability.

Mouse spermatozoa were obtained from the cauda epididymis of control or KD conditional male mice (8 to 13-week-old) and capacitated at 37 °C under 5% CO_2_ for 90 min in a 500 µL drop of Ferticult medium supplemented with 30 mg/mL BSA, under mineral oil.

Cumulus-intact or zona-free oocytes were inseminated for 3 h in a 50 µL drop of medium with capacitated spermatozoa at a final concentration of 1 × 10^6^/mL or 1 × 10^5^/mL, respectively. Then, they were washed and directly mounted in Vectashield/4′, 6-diamidino-2-phenylindole (DAPI) for observation under UV light (Zeiss Axioskop 20 microscope, Big Lake, MN, USA). Were considered fertilized the oocytes showing a fluorescent decondensed sperm head within their cytoplasm.

### 4.7. Statistical Analysis

Results are expressed as mean ± s.e.m. of at least three independent experiments. For statistical analysis, one-way ANOVA multiple comparisons test or t-test were performed using GraphPad Prism version 7.00 for Windows, (GraphPad Software, La Jolla, CA, USA). Differences were considered statistically significant when *p*-value < 0.05.

## Abbreviations

AR	Acrosome reaction
CTRL	control
DAPI	4′, 6-diamidino-2-phenylindole
IVF	In Vitro fertilization
KD	knockdown
KO	knockout
ZP	Zona pellucida
